# Neuroesthetics is Not Just about Art

**DOI:** 10.3389/fnhum.2015.00080

**Published:** 2015-02-17

**Authors:** Dahlia W. Zaidel

**Affiliations:** ^1^University of California Los Angeles, Los Angeles, CA, USA

**Keywords:** esthetics and brain, brain and art, beauty and brain, attention-attraction, biology and esthetics

Esthetics is about beauty. Beauty-generating sources are everywhere around us – in faces, art, food, colors, ideas, nature, and more. An evolutionary approach explains why the brain would not generate esthetic reactions if no useful purpose for survival could be served. Neural processes are strongly selected if they maximize survival. Indeed, from early on, infants aged 2–3 months show esthetic reactions to faces (Langlois et al., 1991; Hoss and Langlois, 2003) and children aged 3 have preferences for friendship based on facial beauty (Dion, 1973). Since adult preferences could not have been learned so early, nor could the concept of beauty itself, this ontogenetic evidence suggests a fundamentally useful biological and adaptive function for esthetics, one that goes beyond enjoyment.

There must have been strong selective pressures to preserve esthetic responses, not only to faces but to a whole range of seemingly unrelated sources as well. The biological roots have been theoretically linked by Charles Darwin in 1871 to sexual selection strategies used by animals to draw attention to healthy, fit mates (typically males) able to mate successfully and produce viable offspring (Darwin, 1981). A great deal of energy is invested in maintaining physical characteristics that maximally showcase health and fitness (Zahavi, 1978; Miller, 2000). The peacock’s tail is a classic such example. To humans, these mating displays are beauty sources that trigger esthetic responses. But it is not a given that the same is elicited in animals. It is proposed here that what may be elicited instead is something akin to attention-attraction to enormous energy investment emphasizing physical and genetic quality. In other words, focused attention with long-term implications is fundamentally at the heart of the display’s purpose. For animals, the end goal of the attention-attraction is selective in that it leads to survival of the species, for humans there is an advantageous outcome of sorts.

Applying the biological origins idea to humans, esthetic responses could be viewed upon as a specialized-type of attraction, a honing in to particular objects, art works, persons, colors, events, ideas, and so on. Whether or not the end goal is related to survival in the way it is with animals may not be the case with all beauty sources, although that this is the case has been suggested by others (Brown et al., 2011). With some sources, the critical benefit of attention-attraction is clear and obvious, as in food coloring (die if we ingest food with the wrong colors), faces (maximize reproductive success if we correctly decipher facial signals), babies’ looks (invest enormous energy in raising and protecting them); with some human-unique sources, such as art, this is less obvious (attraction to the depicted message, to the artistic fitness qualities of the artist), ideas (inspiration for human advancement), and some even less obvious such as scenery of lush foliage or chromatic sunsets (might be related to safe habitat selection).

Given that human brain evolution has sculpted a mind capable of symbolic, abstract, and highly creative cognition, it is reasonable to assume that the original biological animal mating intent of the display has undergone adaptive alterations now expressed as esthetic responses. Otherwise, we would have to assume that esthetic responses factor into animals’ mating choices and that this capacity has been passed on to humans. However, absence of any evidence, or even of current valid empirical methodology, precludes attributing such responses to animals. What is reasonable to assume now is that esthetic reactions in humans reflect a biologically conserved pathway subserving attention-attraction. That is, in the human brain, their purpose has shifted to fit human-unique cognition and existence constraints, while still retaining the adaptive neural mechanism.

But what of art, specifically? It is produced spontaneously only by humans and is ubiquitously practiced throughout the world. It is not immediately obvious how art is necessary or critical for human survival and why it triggers esthetic responses. The early purpose of creating art may not necessarily have been motivated by esthetics, but rather by group membership identification (Lewis-Williams, 2002). The archeological record shows that only minimal and sporadic art was present at the time *Homo sapiens* emerged, around 200,000 years ago, even as human-unique cognition was already evident then and in the preceding thousands of years (McBrearty, 2007). A closer link between art and esthetics could have intensified 45,000–35,000 years ago, a time range when humans began to produce art regularly and prolifically.

The biological notion proposes that whenever art is created it showcases the artist’s genetic qualities such as artistic talent, skill, creativity, and cognitive ability; it is in the brain of the viewer where the esthetic reaction is formed. The argument draws parallels between the energy investment in physiological features used by animals to display their fitness (e.g., the peacock’s long tail) and artists’ mental and physical energy investment in their artwork (weeks, months, and even years on a single piece). The display is the means by which to evaluate the artistic cognitive fitness and the displaying animal’s survival-related genes.

However, looking upon art as a system of communication reveals another key to its practice and value to humans, and therein lies the purpose of the esthetic reaction, namely to pull the viewer in to the artwork through attention-attraction. The benefits of group size increase, generationally retained and transmitted cultural skills (which include art), and social cooperation (Powell et al., 2009) are considered major contributors to expansion and successful development of early *Homo sapiens* (Wadley, 2001; Culotta, 2010). Multi-layered and hierarchical social organization consisting of both kin and non-kin is a critical feature of human society (Hill et al., 2011), and art has become a useful communicative system with social purpose (Luhmann, 2000), not unlike language in this regard. Here, it is proposed that the esthetic reaction to art is actually a neural manifestation of the biological attention-attraction, be it a symbol, figurative or non-figurative art, geometrical, abstract, and so on. Art is currently widely produced for ornamentation, entertainment, conceptual contemplation, cultural and group bonding, propaganda, and much more. Its practice would not have increased and expanded richly into multiple mediums for the past 45,000 years had it not proven to have an adaptive value.

Moreover, the fact that people living far apart nevertheless resonate to art independently of its geographical origin and cultural context reinforces the notion that it is a human communicative system with biological neural origins. An example is that of European artists such as Picasso, Van Gogh, Modigliani, and many others, whose work was influenced by art from Oceania, the Far East, and Africa. Similarly, the fact that we, today, have esthetic reactions to art created in distant prehistoric times, such as cave art, without knowing the context in which it was created, highlights the biological nature of the responses (Zaidel, 2005, 2009).

The neural basis of esthetics has already been established through neuropsychological observations of artists with brain damage (Rose, 2004; Bogousslavsky and Boller, 2005; Zaidel, 2005), physiological brain recordings in healthy subjects making esthetic judgments (for review, Jacobsen, 2013), functional brain neuroimaging of reactions to art works (for review, Nadal, 2013) as well as in esthetic of faces (e.g., Ishai, 2007; Tsukiura and Cabeza, 2011). Discussions of esthetics’ biological roots (Zahavi, 1978; Miller, 2000; Zaidel, 2013) and human brain evolution (for review, Zaidel et al., 2013) have bolstered the link between brain evolution and esthetics.

Beauty is not a distinct and separate component embedded in color, food, nature, scenery, ideas, or even faces. Beauty is not “put in” the artwork by the artist as a distinct entity, but rather it is an emergent property in the brain of the beholder. In addition to its biological pathway and evidence for certain universal preferences, the esthetic reaction is shaped by the viewer’s cultural experiences, life events, education, genetic inheritance, and possibly sex, age, and health status. Individual variability in the intensity and range of the reactions has not been systematically investigated, and much remains to be explored. However, the fact that so many varied sources in human life trigger esthetic responses strongly points to biological, adaptive, and neural underpinning having to do with attention-attraction.

## Conflict of Interest Statement

The author declares that the research was conducted in the absence of any commercial or financial relationships that could be construed as a potential conflict of interest.
